# Risk factors and prognostic predictors for Cervical Cancer patients with lung metastasis

**DOI:** 10.7150/jca.46258

**Published:** 2020-08-08

**Authors:** Xiaoyue Chen, Lan Chen, Haiyan Zhu, Jie Tao

**Affiliations:** 1 Department of Gynecology, Shanghai First Maternity and Infant Hospital, Tongji University School of Medicine, Shanghai 200126, China.; 2 Department of Gynecology, the First Affiliated Hospital of Wenzhou Medical University, Wenzhou 325000, China.

**Keywords:** Cervical cancer, lung metastases, risk factor, prognosis, The Surveillance, Epidemiology, and End Results (SEER)

## Abstract

**Background:** The most common metastatic site in cervical cancers is lung. The aim of this study is to identify cervical cancer patients with high risk for developing lung metastasis and further explore their prognosis.

**Materials and Methods:** At first, patients diagnosed with cervical cancer from 2010 to 2015 were identified from The Surveillance, Epidemiology, and End Results (SEER) database. Multivariate logistic and Cox regression models were used to identify risk and prognostic factors in cervical cancer patients with lung metastasis. Besides, the clinical characteristics of 14 cervical cancer patients followed up for five years with only lung metastases treated at the First Affiliated Hospital of Wenzhou Medical University were retrospectively analyzed.

**Results:** 19,377 cervical cancer patients were selected from the SEER database; the incidence of lung metastases is 4.33%. Multivariable analysis indicated that advantage age (*p<*0.001), non-squamous type (*p*<0.001), late stage (*p*<0.001), lymph nodes metastases (*p*<0.001), and poor differentiation (*p*<0.003) were associated with increased risks for developing lung metastasis. Survival analysis showed that adenocarcinoma, as well as bone and liver metastases were associated with shorter survival in multivariate Cox regression. Among 14 cervical cancer patients with only lung metastasis treated in our hospital, seven patients died within median follow-up time of 16.5 months, including six patients with multiple lung metastasis lesions and one patient with solitary lesion. Seven patients received pulmonary metastasectomy and the following cisplatin-based chemotherapy, among whom one patient died during follow up.

**Conclusions:** Lung metastasis has poor prognosis. Senior age, non-squamous type, late stage, lymph nodes metastases, and poor differentiation are associated with an increased risk for lung metastasis. We recommend pulmonary imaging assessment within 2 years after primary treatment. Adenocarcinoma and multiple pulmonary lesions might predict poor prognosis. To those patients with resectable lung metastasis lesion and no other organ involvement, metastasectomy might improve survival.

## Introduction

Carcinoma of the uterine cervix remains a leading cause of cancer-related mortality in women, causing 311,365 deaths in 2018 worldwide 1. Despite early-stage or locally advanced cervical cancer patients that have a good prognosis because of appropriate therapy, metastatic cervical cancer remains lethal with a median survival of 8-13 months, with no standard treatment 2, 3.

Pulmonary metastasis is the most common metastasis in cervical cancer 4, 5. Lung metastasis in cervical cancer is attributed to hematogenous spread. The reported incidence of lung metastasis in cervical cancer ranges from 4.16% to 7.7% 3, 6, 7. Most patients are asymptomatic and detect on routine chest X-ray or computed tomography at outpatient follow-up visit 6, 8. Therefore, identifying patients with high risk for lung metastases and taking early intervention are important to gynecological oncologist.

Treatment for cervical cancer patients with lung metastasis includes surgical resection, chemotherapy, or radiotherapy. The median survival time is 18 months 9. The clinical characteristics of cervical cancer patients with lung metastasis remain poorly investigated because of its low prevalence and the lack of large-scale population-based study. In this study, we analyzed the incidence, the risk and prognosis factors for lung metastases from cervical cancer using the Surveillance, Epidemiology, and End Results (SEER) database 10. Meanwhile we retrospectively reviewed the files of 14 patients with only lung metastases from cervical carcinoma treated in our hospital to analyze their clinical characteristics, treatments, and outcomes.

## Materials and Methods

### SEER Database

**Figure 1:**
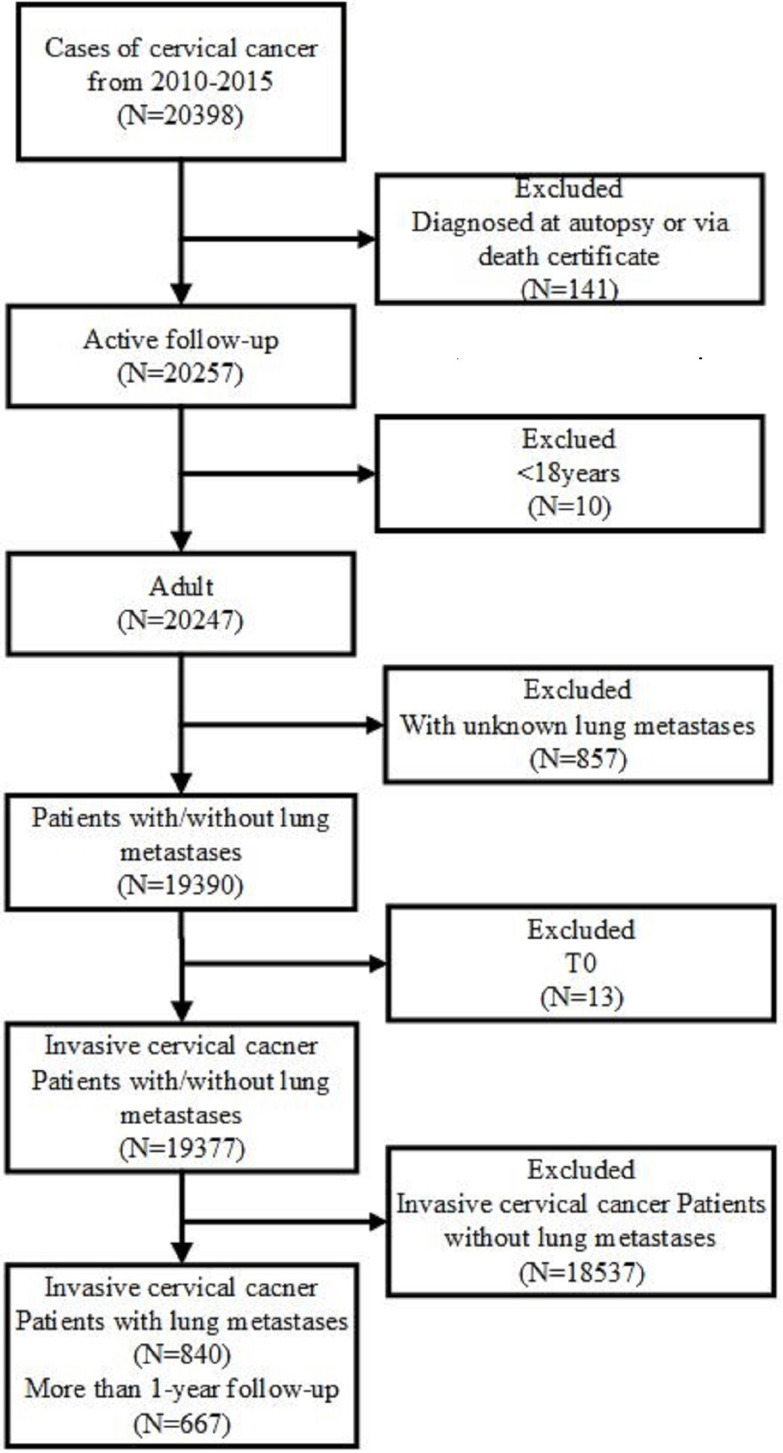
Flowchart of the patient's enrollment in this study according to the inclusion and exclusion criteria.

Public original data were obtained from the SEER database. Data were downloaded by SEER*Stat Software version 8.3.5 (https://seer.cancer.gov/data/) (National Cancer Institute, Bethesda, MD, USA). The inclusion criteria included primary site code ICD-O-3 (International Classification of Diseases for Oncology-3)/WHO 2008 restricted to 'cervix uteri', and the diagnosis was made from January 1, 2010 to December 31, 2015. The exclusion criteria were listed as follows: (1) patients diagnosed at autopsy or via death certificates; (2) the age at diagnosis younger than 18 years; (3) unknown lung metastases; (4) in-situ, benign or borderline tumors. Besides, patients who had unclear TNM stage record, unknown survival time, missing cause of death, or unknown diagnostic confirmation were subsequently excluded (Figure 1).

Records from SEER between January 1, 2010 and December 31, 2015 were included in the analysis of the incidence and risk factors for lung metastases. Those records diagnosed from January 1, 2010 to December 31, 2014 (with at least one-year follow-up) were included to perform survival analysis and to investigate the prognostic factors for lung metastases. Those records diagnosed from January 1, 2010 to December 31, 2014 were included in the data from January 1, 2010 to December 31, 2015, so their clinical parameter was supplied as Supplementary material in Table S1.

We stratified the cohort by age and marital status at diagnosis, race, insurance status, TNM (Tumor, Node and Metastasis) staging, histological types, pathological grade, surgery and the presence of other distant site metastasis (liver, brain and bone).

### Patient data from our hospital

Invasive cervical cancer patients with lung metastasis after radical hysterectomy between January 2008 and December 2018 at the First Affiliated Hospital of Wenzhou Medical University, China, were included in our study. To eliminate the influence of late stage related multiple organ metastasis, we choose early stage cervical cancer patients (stage I-II) with lung metastasis as the research objects. The inclusion criteria are Stage I-II cervical cancer, lung metastasis found after hysterectomy in our hospital, but no distant metastasis in other organs. CT was the method of confirming lung metastases in patients with cervical cancer and the follow up time is five years.

This study complied with the Declaration of Helsinki and followed the ethical principles of the First Affiliated Hospital of Wenzhou Medical University. Patients had signed written informed consents to be included in the study. Clinical and pathologic information were obtained from patient files and pathology reports.

The median age of all patients is 46.5 (range from 37 to 63). Four cases are in Stage I, and ten in Stage II according to 2009 FIGO staging criteria. Ten patients are diagnosed squamous cell cancer, three adenocarcinoma and one clear cell cancer. 90.9% of the patients have deep stromal invasion. Four cases have pelvic lymph node metastasis.

### Statistical analysis

SPSS version 13.0 (IBM Inc., Chicago, IL, USA) was used for statistical analysis, and survival curves were generated using GraphPad Prism version 6.0 (GraphPad-Prism Software Inc., San Diego, CA, USA). Categorical variables were shown as number (percentage). Association between categorical data was analyzed using the chi-squared test or rank sum test. Univariate logistic regression analysis was performed to select risk factors for lung metastases and those with *p*<0.05 were then further analyzed using a multivariate logistic regression model. The Kaplan-Meier curves and Cox regression analysis were applied to determine prognostic factors associated with overall or cancer-specific survival A *p*-value <0.05 was considered as statistically significant.

## Results

### Clinical characteristics in SEER Cohort

Totally 19,377 cervical cancer patients diagnosed from 2010 to 2015 met the inclusion criteria, among them 840 patients (4.33%) had lung metastases, and 18,537 patients did not. The mean age was 51.35±14.98 years. The clinical characteristics of patients with or without lung metastasis were presented in Table 1. Comparing to patients without lung metastases, those suffered lung metastasis were more likely to accompany with lymph node metastases (N1: 55.83% vs 23.49%, *p*<0.001), poor-differentiated (G3: 42.74% vs 27.79%,* p*<0.001), extracercical extension (T3: 38.33% vs 14.67%,* p*<0.001), and without surgical treatment of primary site (91.19% vs 42.21%,* p*<0.001).

### Risk factors for developing lung metastases in SEER Cohort

**Figure 2:**
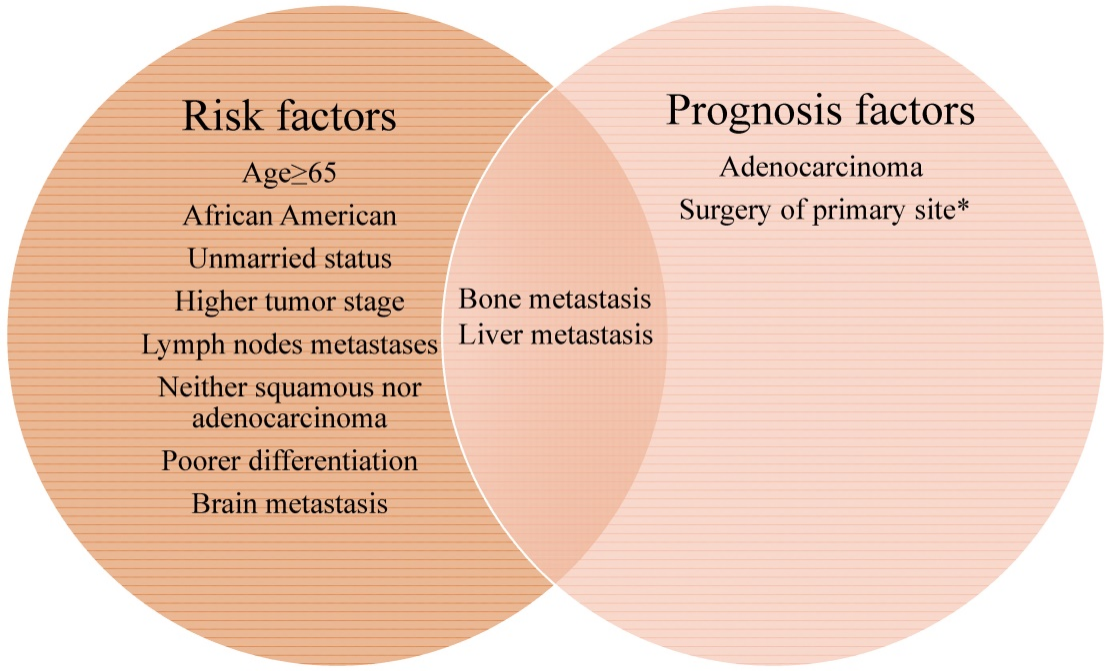
The Venn diagram of risk and prognosis factors of cervical cancer lung metastasis. All the factors included in the left circle represent the risk factors for developing lung metastasis. The factors included in the right circle were associated with mortality. Bone and liver metastasis are both the risk and prognosis factors for lung metastasis in cervical cancer patients. *The median cancer specific survival time was prolonged from 15.95 (95% CI: 14.00-17.90) months to 30.70 (95% CI: 21.24-40.15) months in patients with surgery of primary site.

We then clarified the risk factors significantly associated with the lung metastases among patients with invasive cervical cancer. As depicted in Table 2, univariate analysis showed that senior citizens, African American, unmarried status, higher tumor stage, lymph nodes metastases, special (neither squamous nor adenocarcinoma) histological types, poor differentiation, the presence of bone, brain and liver metastases, and without surgical treatment of primary site were correlated with higher risk for lung metastases. As was shown in Figure 2, all the factors included in the left circle represent the risk factors for developing lung metastasis. Further multivariable logistic regression analysis indicated that advanced age, higher tumor stage, positive lymph nodes, special histological types, poor differentiation, surgical treatment of primary site and others site metastasis (brain, bone and liver) were positively associated with lung metastases (Table 2).

### Survival outcomes and prognostic factors for lung metastases in SEER Cohort

**Figure 3:**
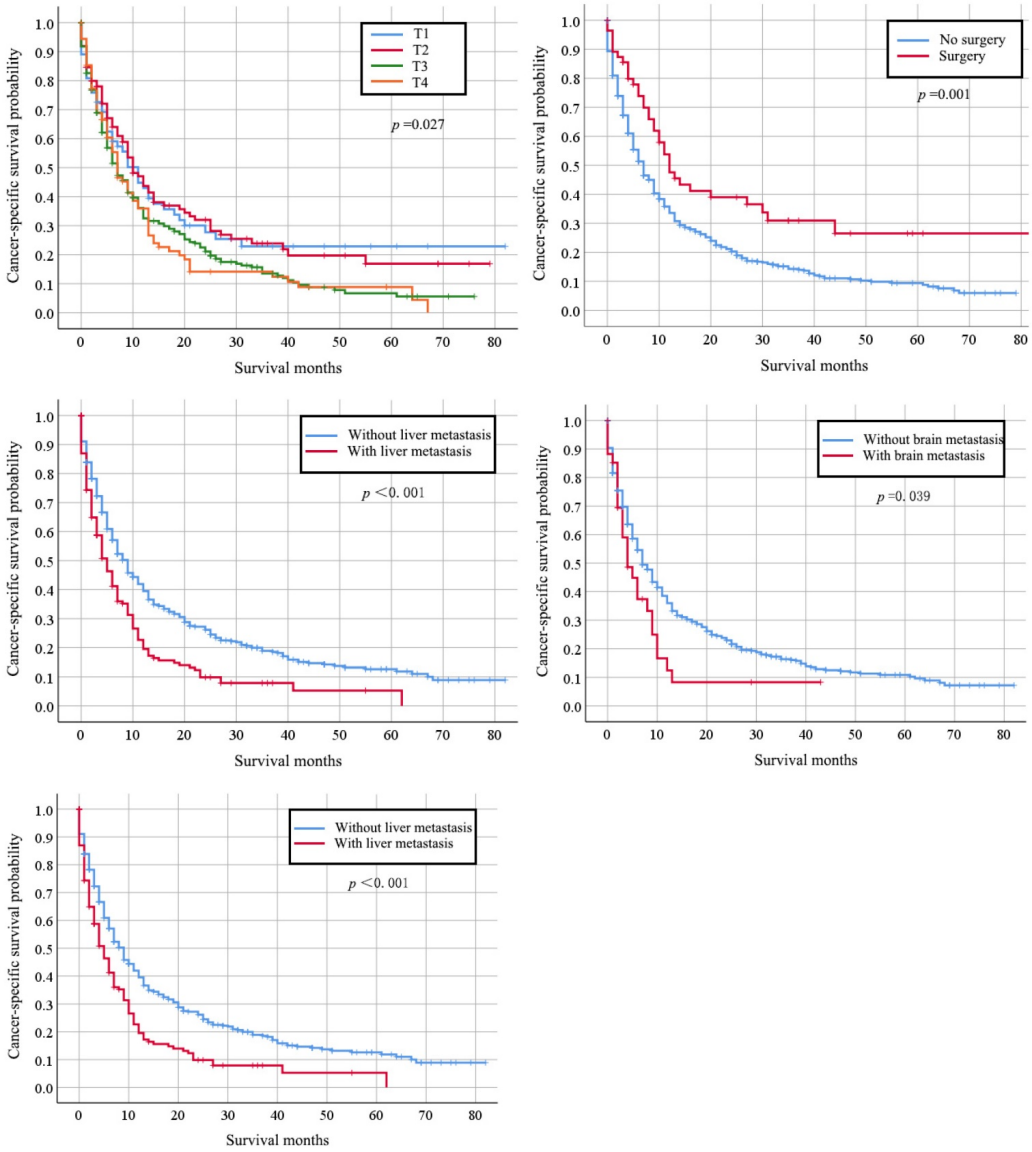
Kaplan-Meier analysis of overall survival in cervical cancer patients with lung metastasis stratified by age at diagnosis, grade, stage, primary surgery and other organs metastasis.

**Figure 4:**
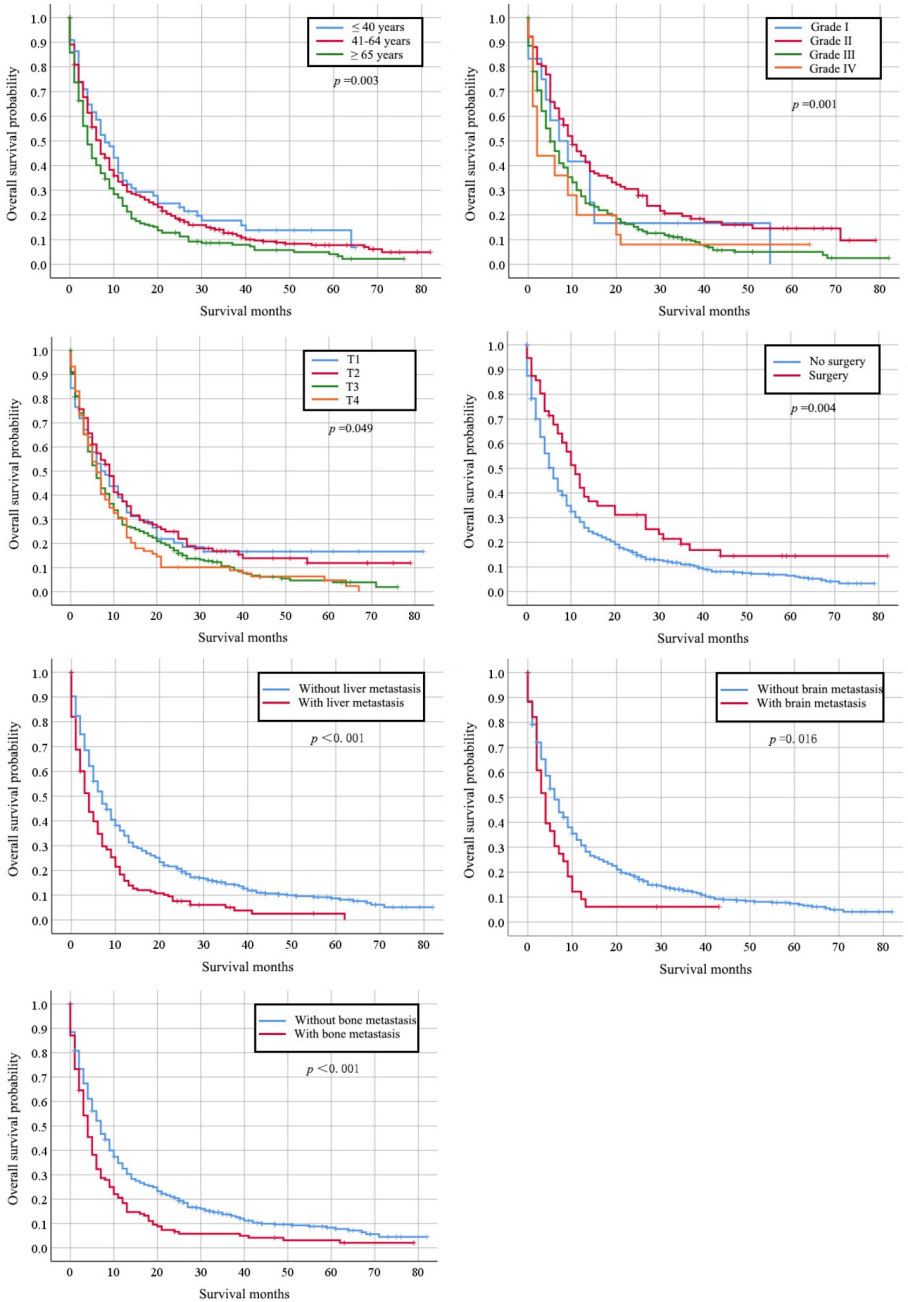
Kaplan-Meier analysis of cancer-specific survival in cervical cancer patients with lung metastasis stratified by stage, primary surgery and other organs metastasis.

Totally, 667 cervical cancer patients who had lung metastasis underwent follow-up for at least one year were included for survival analysis. Their median survival time is 6 months (95%CI: 5.22-6.78). The survival rate is 29.30% (one-year), 17.6% (two-year), and 7% (five-year) respectively. Their clinical characteristics are shown in Table S1. Those records diagnosed from January 1, 2010 to December 31, 2014 were included in the data from January 1, 2010 to December 31, 2015, so that detailed description of the clinical characters was not go into details. Multivariate Cox regression indicated that histology type of adenocarcinoma (HR: 0.745, 95% CI: 0.564-0.983, *p*=0.038), bone metastases (HR: 1.340, 95% CI: 1.036-1.731, *p*=0.025), and liver metastases (HR: 1.705, 95% CI: 1.331-2.184, *p <* 0.001) were independently negative prognosis factors for overall survival (Table 3). Similarly, these factors were also associated with shorter cancer-specific survival (Table S2). The median cancer specific survival time was prolonged from 15.95 (95% CI: 14.00-17.90) months to 30.70 (95% CI: 21.24-40.15) months in patients with surgery of primary site (Figure 2). Kaplan-Meier analysis of overall survival (Figure 3) and cancer-specific survival (Figure 4) in cervical cancer patients with lung metastasis were also performed. Age and surgery were statistically significant associated with overall survival.

### Clinical characteristics of patients with only lung metastasis in our center

We depicted the clinical characteristics and treatment of the 14 cervical cancer patients with only lung metastasis from our hospital. The detailed clinical information was shown in Table 4. The median interval between initial treatment and onset of lung metastasis was 21 months (range from 7 to 35 months). Ten cases suffered metastasis within 2 years after surgery. Of all the 14 cases, seven patients have solitary lung metastatic lesions, and the others have multiple lung metastatic lesions.

Seven deaths were occurred among these 14 patients with a median follow-up time of 16.5 months (from pulmonary metastasis to death or last follow-up). In those patients died during our study, six patients have multiple lung metastasis lesions and only one patient has solitary lung metastasis lesion. Patients with solitary lesion had longer medium interval between lung metastasis and death or last follow-up (33.29 months vs 16.43 months, *p*<0.05). When it comes to the regimen, among the seven patients received pulmonary metastasectomy and the following cisplatin-based chemotherapy, only one patient died because of the disease. Five patients took cisplatin-based chemotherapy, one patient underwent chemoradiotherapy and one patient rejected any treatment.

## Discussion

Metastatic cervical cancer remains a major cause of cancer related death in women. Lung is the most common metastatic organ, which accounts almost 50% of all metastasis 4, 11. We found in this study that the incidence of lung metastasis for cervical cancer was 4.33%, which is consistent with previous reports 6, 8, 12. The prognosis of cervical cancer patients with lung metastasis is far from satisfactory.

Since cervical cancer with lung metastases are usually asymptomatic and present poor prognosis, there is a crying need to identify patients with high-risk for lung metastasis. In this study, we found that patients with age greater than 65, non-squamous histology type, late stage, pelvic lymph nodes metastases, poor differentiation, other organ metastasis and without operation at the first treatment were more likely to suffer lung metastasis. It is not surprising that patients with advanced disease, non-squamous type, and lymphatic metastasis are more likely to suffer lung metastasis, since these factors have been well described as poor prognostic predictors for cervical cancer 13, 14. Ageing has been recognized as the biggest risk factor for cancer because of the accumulation of mutations and compromised immune system 15. Cervical cancer is no exception. The incidence of cervical cancer among all women with an intact cervix continues to increase through at least age 69 years 16, 17. It is reported that elder women with cervical cancer had a poor overall survival without the influence of stages and histologic subtypes 18, 19. We found in our study that the odds risk was increasing with age, which further support the opinion that age is an independent negative prognostic factor for cervical cancer. Poor differentiated tumor cells are usually more aggressive, which might partially explain our results that poor differentiation was significantly linked to higher risk of lung metastasis 20.

There are two metastasis approaches for cervical cancer, hematogenous and lymphatic spread. The most common organs of cervical cancer metastasis are lung, bone, liver, and brain. Our current results showed that the coexistence of lung and other organs (bone, liver, and brain) metastasis was common, as they were all caused by hematogenous spread 21. Accordingly, we suggest that those patients with senior age, special histologic type, lymph nodes metastasis as well as poor differentiation should take pulmonary imaging assessment to detect early lung metastasis.

Survival analysis was also performed. We found the accompanied bone or liver metastasis might indicate poor prognosis to cervical cancer patients with lung metastases. Our results revealed that patients with multiple organ metastases presented worse prognosis than those with solitary organ metastases. With respect to pathological features, most authors reported that adenocarcinoma had a greater propensity to lymph node and distant metastases. We found adenocarcinoma was the only independent unfavorable prognostic variable for patients with lung metastases, which was the same as the results from others 22 23. It is reported that patients with adenocarcinoma and adenosquamous carcinoma tended to have a significantly lower 5-year disease free survival when comparing with squamous cell carcinomas (0% VS 47.4%) 23. The poor prognosis of adenocarcinoma and adenosquamous carcinoma might be explained by those biological variables still under investigation, such as cyclin-dependent kinase inhibitors, p53, cyclooxygenase-2 (COX-2), cell surface tyrosine-kinases and programmed death-ligand (PD-L1) 24. Cisplatin-based palliative chemotherapy is the only treatment option for recurrent or metastatic cervical cancer intolerance to surgery and radiotherapy 25. We speculated that the poor prognosis of adenocarcinoma and adenosquamous carcinoma might be related to their insensitivity to chemoradiation. Adenocarcinomas are less sensitive to radiation than squamous-cell carcinomas 26, 27. Although adenocarcinoma of the ovary or endometrium is often sensitive to chemotherapy, adenocarcinomas of the cervix are still lack of sensitive chemotherapy regimens 28. It was reported that cervical cancer patients with non-squamous cell carcinoma were more likely to have lung metastasis 29. ERBB2 mutations concomitantly harbored PIK3CA or KRAS mutations were found in non-squamous cervical cancer 30. The molecular difference between non-squamous cell carcinoma and squamous cell carcinoma might partially be explained by their biological behavior.

Finally, we summarized the clinical characteristics and antineoplastic treatment of 14 patients with only lung metastasis from our hospital. We found that the median interval between initial treatment and onset of lung metastasis was 21 months (range from 7 to 35 months). Ten cases suffered metastasis within 2 years after surgery. Our results are consistent with other studies. It was reported that 58% percent of the recurrences were observed within the first 12 months after surgery and 83% within the first 2 years 31, and the mean event-free duration (from initial treatment to relapse or metastasis) was 12 months 6 and 24 months 31, 32. Since many patients with pulmonary metastasis have no specific symptoms, serological tumor markers, such as SCC 33, 34, can detect tumor recurrence, but it is not specific for lung metastasis, routine follow-up chest X-ray or CT might be helpful for early detection of lung metastasis. Among the seven patients with multiple lung metastasis lesions, six patients died. While in those with solitary lung metastasis lesions, only one patient passed away. Patients with multiple lung metastasis lesions are more likely to have poor prognosis, and it is consistent with other related studies. Yamamoto et al. reported that patients with one or two metastatic pulmonary foci showed a higher 5-year survival rate than patients with three or four metastatic pulmonary foci 23. Ki et al reported that the overall survival of patients with no more than 3 metastatic lung lesions are longer than those with more than 4 lesions 6.

Currently, there is no standard treatment option for patients with lung metastases from cervical cancer. Systemic treatment with chemotherapy is the main treatment. But it has no improvement on the poor median survival, which is reported to be 0.69 year 3. Surgical resection of the pulmonary lesion was an effective treatment for those patients without lesions in other organs 22, 35. In our study, seven patients received lobectomy, six cases are still alive. Our observation further supports the therapeutic effectiveness of pulmonary lesion resection in cervical cancer with resectable lung lesion. According to the published report and our experience, we recommended that patients with resectable lung metastases from cervical cancer underwent metastasectomy.

Our study had some limitations. Firstly, it is a retrospective study, and it might have some bias which could not be avoided. Secondly, the incidence of lung metastases might be underestimated. Thirdly, we acknowledged the limitations due to the limited sample size. We are collecting data for more patients in our hospital as well as cooperated with another institution; and will update our study in the future.

To sum up, lung metastasis from cervical cancer is rare and has a poor prognosis. Advantage age, non-squamous histology subtype, late stage, lymph nodes metastases and poor differentiation are associated with an increased risk for developing lung metastasis. Pulmonary imaging assessment is highly recommended to these patients within 2 years after primary treatment. Adenocarcinoma and multiple pulmonary lesions are negative prognostic factor for cervical cancer patients with lung metastasis. Metastasectomy might be helpful to prolong survival, especially to those patients with solitary lung lesion and without other organs involved.

## Supplementary Material

Supplementary figures and tables.

## Figures and Tables

**Table 1:** Clinical characteristics for patients diagnosed invasive cervical cancer with and without lung metastasis in SEER database (2010-2015)

Subject characteristics	No. of invasive cervical cancer patients (2010-2015)		χ^2^	*P* value
	Lung metastasis, N=840 (4%)	Without lung metastasis, N=18537 (96%)		
**Age (years)**			182.918	<0.001
≤40	86 (10.24%)	5286 (28.52%)		
40-64	483 (57.50%)	9864 (53.21%)		
≥65	271 (32.26%)	3387 (18.27%)		
**Ethnicity**			15.102	0.002
White	620 (73.81%)	13818 (75.54%)		
Black	141 (16.79%)	2558 (13.80%)		
Others	79 (9.40%)	1967 (10.61%)		
Unknown	0 (0.00%)	194 (1.05%)		
**Marital status**			12.092	0.002
Married	299 (35.60%)	7596 (40.98%)		
Unmarried	494 (58.81%)	9767 (52.69%)		
Unknown	47 (5.59%)	1174 (6.33%)		
**Insurance status**			10.644	0.005
Insured	464 (55.24%)	11256 (60.72%)		
Uninsured	57 (6.78%)	1202 (6.48%)		
Unknown	319 (37.98%)	6079 (32.79%)		
**T-stage**			1291.477	<0.001
T1	89 (10.60%)	10366 (55.92%)		
T2	143 (17.02%)	4095 (22.09%)		
T3	322 (38.33%)	2720 (14.67%)		
T4	112 (13.33%)	612 (3.30%)		
Unknown	174 (20.71%)	744 (4.01%)		
**N-stage**			910.132	<0.001
N0	222 (26.43%)	13442 (72.51%)		
N1	469 (55.83%)	4354 (23.49%)		
Unknown	149 (17.74%)	741 (4.00%)		
**Histology**			86.426	<0.001
Squamous	498 (59.29%)	12111 (65.33%)		
Adenocarcinoma*	157 (18.69%)	4298 (23.19%)		
Others	185 (22.02%)	2128 (11.48%)		
**Grade**			18629.047	<0.001
G1	16 (1.90%)	2128 (11.48%)		
G2	149 (17.74%)	5842 (31.52%)		
G3	359 (42.74%)	5152 (27.79%)		
G4	36 (4.29%)	408 (2.20%)		
Unknown	280 (33.33%)	5007 (27.01%)		
**Bone metastasis**			1778.328	<0.001
None	632 (75.24%)	18256 (98.48%)		
Yes	187 (22.26%)	263 (1.42%)		
Unknown	21 (2.50%)	18 (0.10%)		
**Liver metastasis**			2289.750	<0.001
None	620 (73.81%)	18321 (98.83%)		
Yes	204 (24.29%)	203 (1.10%)		
Unknown	16 (1.90%)	13 (0.07%)		
**Brain metastasis**			813.769	<0.001
None	773 (92.02%)	18489 (99.74%)		
Yes	45 (5.56%)	35 (0.19%)		
Unknown	22 (2.62%)	13 (0.07%)		
**Surg (Prim)**			781.690	<0.001
None	766 (91.19%)	7824 (42.21%)		
Yes	73 (8.69%)	10680 (57.61%)		
Unknown	1 (0.12%)	33 (0.18%)		

*Including adenosquamous; Surg (prim) = surgical treatment of primary site.

**Table 2:** Univariate and multivariate logistic regression analysis for the associated risk factors for developing lung metastases in patients diagnosed with cervical cancer between 2010-2015

Subject characteristics	Univariate analysis		Multivariate analysis	
	OR (95% CI)	*P*-value	OR (95% CI)	*P*-value
**Age (years)**				
<40	Reference	1.000	Reference	1.000
40-64	3.010 (2.387-3.795)	<0.001	2.357 (1.349-4.117)	0.003
≥65	4.918 (3.844-6.292)	<0.001	3.485 (1.945-6.243)	<0.001
**Ethnicity**				
White	Reference	1.000	Reference	1.000
Black	1.228 (1.018-1.482)	0.032	0.882 (0.586-1.327)	0.546
Others	0.895 (0.705-1.137)	0.363	0.859 (0.510-1.447)	0.568
Unknown	NA	NA	NA	NA
**Marital status**				
Married	Reference	1.000	Reference	1.000
Unmarried	1.285 (1.110-1.488)	0.001	1.025 (0.766-1.372)	0.867
Unknown	NA	NA	NA	NA
**T-stage**				
T1	Reference	1.000	Reference	1.000
T2	4.067 (3.114-5.312)	<0.001	1.479 (0.920-2.377)	0.106
T3	13.788 (10.863-17.502)	<0.001	3.592 (2.290-5.635)	<0.001
T4	21.315 (15.949-28.487)	<0.001	4.053 (2.329-7.053)	<0.001
**N-stage**				
N0	Reference	1.000	Reference	1.000
N1	6.522 (5.540-7.679)	<0.001	2.556 (1.894-3.451)	<0.001
Unknown	NA	NA	NA	NA
**Histology**				
Squamous	Reference	1.000	Reference	1.000
Adenocarcinoma	0.888 (0.740-1.066)	0.204	1.882 (1.328-2.666)	<0.001
Others	2.114 (1.775-2.518)	<0.001	1.664 (1.092-2.534)	0.018
**Grade**				
G1	Reference	1.000	Reference	1.000
G2	3.392 (2.021-5.694)	<0.001	2.172 (0.945-4.988)	0.068
G3	9.268 (5.602-15.331)	<0.001	3.373 (1.496-7.605)	0.003
G4	11.735 (6.454-21.348)	<0.001	2.818 (1.046-7.591)	0.041
Unknown	NA	NA	NA	NA
**Bone metastases**				
None	Reference	1.000	Reference	1.000
Yes	20.539 (16.756-25.176)	<0.001	3.894 (2.539-5.973)	<0.001
Unknown	NA	NA	NA	NA
**Liver metastases**				
None	Reference	1.000	Reference	1.000
Yes	29.696 (24.067-36.640)	<0.001	7.925 (5.062-12.407)	<0.001
Unknown	NA	NA	NA	NA
**Brain metastases**				
None	Reference	1.000	Reference	1.000
Yes	30.752 (19.657-48.111)	<0.001	4.215 (1.531-11.607)	0.005
Unknown	NA	NA	NA	NA
**Surg (Prim)**				
None	Reference	1.000	Reference	1.000
Yes	0.070 (0.055-0.089)	<0.001	0.276 (0.180-0.424)	<0.001
Unknown	NA	NA	NA	NA

NA= not available, Surg (Prim) = surgical treatment of primary site.

**Table 3:** Multivariate Cox regression analysis of overall survival in cervical cancer patients with lung metastases in SEER database (2010-2015)

Subject characteristics	Overall Survival, Median (IQR), months	HR (95% CI)	*P*-value
**Age (years)**			
<40	17.228 (12.027-22.430)	Reference	1.000
40-64	15.217 (12.994-17.440)	1.142 (0.789-1.653)	0.483
≥65	10.630 (8.404-8.404)	1.481 (0.995-2.204)	0.053
**Grade**			
G1	15.083 (4.092-26.075)	Reference	1.000
G2	21.388 (16.617-26.159)	0.685 (0.363-1.294)	0.244
G3	12.463 (10.302-14.625)	0.992 (0.535-1.838)	0.979
G4	10.320 (3.634-17.006)	1.325 (0.634-2.770)	0.455
Unknown	NA	NA	NA
**Histology**			
Squamous	12.942 (11.136-14.747)	Reference	1.000
Adenocarcinoma	17.304 (13.072-21.537)	0.745 (0.564-0.983)	0.038
Others	13.264 (9.838-16.691)	0.816 (0.615-1.082)	0.158
**Bone metastases**			
None	15.568 (13.619-17.517)	Reference	1.000
Yes	8.657 (6.225-11.088)	1.340 (1.036-1.731)	0.025
Unknown	NA	NA	NA
**Liver metastases**			
None	16.090 (14.066-18.114)	Reference	1.000
Yes	7.920 (5.974-9.866)	1.705 (1.331-2.184)	<0.001
Unknown	NA	NA	NA
**Brain metastases**			
None	14.474 (12.777-16.170)	Reference	1.000
Yes	6.755 (3.382-10.127)	1.513 (0.969-2.362)	0.069
Unknown	NA	NA	NA
**Surg (Prim)**			
None	13.186 (11.603-14.768)	Reference	1.000
Yes	22.423 (15.219-29.627)	0.699 (0.485-1.007)	0.054
Unknown	NA	NA	NA

Sur(prim) = surgical treatments of primary site, NA = Not available, all factors with Unknown data were removed from Cox and Kaplan-Meier model.

**Table 4:** Clinical and pathological characteristics, treatment modalities, and outcome

Patient	Age at Diagnosis	Histology	FIGO Stage	Pelvic LN Involvement	Lymphovascular space invasion	HPV Infection	Number of pulmonary metastases	Interval between operation and lung metastasis (months)	Treatment after lung metastasis	Interval between lung metastasis and death or last follow-up (months)	Status at last follow up
1	48	Adeno-carcinoma	IIa2	No	No	NA	Multiple	25	Chemotherapy	17	DOD
2	51	Squamous carcinoma	IIa1	Yes	Yes	16	Multiple	10	Chemotherapy	3	DOD
3	45	Adeno-carcinoma	IIa2	No	No	Negative	Multiple	17	Untreated	5	Alive
4	60	Clear cell	Ib1	Yes	Yes	NA	Single	19	Lobectomy+chemotherapy	24	DOD
5	45	Squamous carcinoma	Ib1	No	No	16,39	Multiple	24	Lobectomy+chemotherapy	16	DOD
6	63	Squamous carcinoma	Ib1	No	No	16,53	Multiple	35	chemoradiotherapy	11	DOD
7	57	Squamous carcinoma	IIa1	No	Yes	16,52	Multiple	9	Chemotherapy	37	DOD
8	37	Squamous carcinoma	Ib1	No	Yes	16	Single	7	Lobectomy+chemotherapy	44	Alive NED
9	42	Squamous carcinoma	IIa1	Yes	Yes	16	Single	10	Lobectomy+chemotherapy	12	Alive NED
10	56	Adeno-carcinoma	IIa2	Yes	Yes	NA	Single	28	Lobectomy+chemotherapy	22	Alive NED
11	39	Squamous carcinoma	IIa1	No	No	16	Single	10	Chemotherapy	20	Alive
12	60	Squamous carcinoma	IIa1	No	No	NA	Single	24	Lobectomy+chemotherapy	84	Alive NED
13	39	Squamous carcinoma	IIa2	No	IIa2	Negative	Multiple	23	Chemotherapy	26	DOD
14	44	Squamous carcinoma	IIa1	No	IIa1	NA	Single	32.5	Lobectomy+chemotherapy	27	Alive NED

LN, lymph node; NA, not available; DOD, die of disease; NED, no evidence of disease.
