# Supplementary material for: Risk factors and prognostic predictors for Cervical Cancer patients with lung metastasis
Source: J Cancer. 2020 Aug 8;11(20):5880–9. doi: 10.7150/jca.46258 (PMC7477410; doi:10.7150/jca.46258)
Supplement: Supplementary file 1 — Supplementary figures and tables. [file jcav11p5880s1.pdf]

Table S1. Clinical characteristics for patients diagnosed invasive cervical cancer with and without lung metastasis in SEER database (2010-2014).

Subject characteristics	No. of invasive cervical cancer patients (2010–2014)	
	lung metastases (N=667)	Without lung metastases (N=15349)
Age(years)		
≤40	66(9.90%)	4385(28.57%)
40-64	383(57.42%)	8154(53.12%)
≥65	218(32.68%)	2810(18.31%)
Ethnicity		
White	486(72.86%)	11438(74.52%)
Black	115(17.24%)	2153(14.03%)
Others	66(9.90%)	1612(10.50%)
Unknown	0(0.00%)	146(0.95%)
Marital status		
Married	231(34.63%)	6302((41.06%)
Unmarried	401(60.12%)	8074(52.60%)
Unknown	35(5.25%)	973(6.34%)
Insurance status		
Insured	51(7.65%)	1052(6.85%)
Uninsured	360(53.97%)	9250(60.26%)
Unknown	256(38.38%)	5047(32.88%)
T-stage		
T1	64(9.60%)	8610(56.09%)
T2	111(16.64%)	3371(21.96%)
T3	267(40.03%)	2250(14.66%)
T4	89(13.34%)	501(3.26%)
Unknown	136(20.39%)	617(4.02%)
N-stage		
N0	172(25.79%)	11190(72.90%)
N1	370(55.47%)	3547(23.11%)
Unknown	125(18.74%)	612(3.99%)
Histology		
Squamous	392(58.77%)	10045(65.44%)
Adenocarcinoma*	122(18.29%)	3552(23.14%)
Others	153(22.94%)	1752(11.41%)
Grade		
G1	12(3.24%)	1749(11.39%)
G2	117(17.54%)	4840(31.53%)
G3	289(43.33%)	4280(27.88%)
G4	25(3.75%)	327(2.13%)
Unknown	224(33.58%)	4153(27.06%)
Bone metastasis		

None	512(76.76%)	15130(98.57%)
Yes	139(20.84%)	205(1.34%)
Unknown	16(2.40%)	14(1.04%)
Liver metastasis		
None	493(73.91%)	15168(98.82%)
Yes	161(24.14%)	169(1.10%)
Unknown	13(1.95%)	12(0.08%)
Brain metastasis		
None	614(92.05%)	15310(99.75%)
Yes	34(5.10%)	29(0.19%)
Unknown	19(2.85%)	10(0.07%)
Surg (Prim)		
None	610(91.45%)	6435(41.92%)
Yes	56(8.40%)	8884(57.88%)
Unknown	1(0.15%)	30(0.20%)

* Including adenosquamous; Surg (prim)=surgical treatment of primary site.

Table S2. Multivariate Cox regression analysis of cancer-specific death in cervical cancer patients with lung metastases in SEER database (2010–2014)

Subject characteristics	Survival, Median (IQR), months	HR (95% CI)	*p*-value
Age(years)			
<40	18.966(13.348-24.585)	Reference	1.000
40-64	17.565(14.952-20.177)	1.099(0.750-1.611)	0.627
≥65	15.428(12.012-18.843)	1.242(0.817-1.887)	0.310
Grade			
I	23.108(6.987-39.228)	Reference	1.000
II	24.746(19.247-30.245)	0.974(0.446-2.128)	0.947
III	14.820(12.188-17.451)	1.361(0.633-2.925)	0.429
IV	15.240(5.747-24.733)	1.821(0.755-4.391)	0.182
Unknown	NA	NA	NA
Histology			
Squamous	15.393(13.179-17.606)	Reference	1.000
Adenocarcinoma	22.208(16.777-27.639)	0.709(0.522-0.963)	0.027
Others	16.581(12.336-20.825)	0.845(0.627-1.138)	0.267
Bone mets			
None	19.289(16.831-21.746)	Reference	1.000
Yes	9.788(7.016-12.559)	1.423(1.087-1.864)	0.010
Unknown	NA	NA	NA
Liver mets			
None	19.576(17.083-22.069)	Reference	1.000
Yes	10.031(7.389-12.674)	1.635(1.253-2.134)	＜0.001
Unknown	NA	NA	NA
Brain mets			
None	17.669(15.557-19.781)	Reference	1.000
Yes	8.231(3.980-12.482)	1.449(0.900-2.333)	0.127
Unknown	NA	NA	NA
Surg (Prim)			
None	15.950(14.000-17.899)	Reference	1.000
Yes	30.696(21.238-40.154)	0.616(0.406-0.934)	0.022
Unknown	NA	NA	NA
